# Supplementary material for: Codon choice directs constitutive mRNA levels in trypanosomes
Source: eLife. 2018 Mar 15;7:e32467. doi: 10.7554/eLife.32467 (PMC5896880; doi:10.7554/eLife.32467)
Supplement: Supplementary file 1. — Each sequence is split into three: the 5’ sequences, the open reading frame in bold, and 3’ sequences. [file elife-32467-supp1.docx]

**p3827 beta tubulin 5’- MS2bp-eGFP-nls ORF – actin 3’**

TTAATTAATAGACGCGGACGGGGCATTTCCCGTTCGTCATTAGCAGTAGGTAATGAAGATGTTTGTTTCTCGTCCCCTTTCTCCTTCGTCCTTCTGTCATTTTGTTCTTTTGTGTTTATGTTTTGTTGTTGTTTTCTTTAATTTTTTTTTTTCTTCCACGTTTGTGTACATCCGCGCGCCACTCTATTCAGAGAGCCACGGATAGTAGAGGAGGTGGGAAGGGTATATGAGGGACACGCGTACCATGATGTGGGATGTATTGGGGTCCCTGTCTGTCCTTACGTGACTATGTATGAACCGTCACGTGTAAGATGAGCTAGTGAGATCAACAGTACAACTCATCAACACGCCTTCTTCTCGTTAAATGTACACAATCTTGATCCTCCACCTTTATGGGTCCCATTGTTTGCCTCTTCCGCTGTGTGGAGTGCGCCTACACGCACTTCTCACTTCGTAAGTGGTGGTGGCGTAAGTATTGCCTAATGTTGACTCTATATTCTCCTCTCCTCACCCCCTCGCGGTGCTGATTTCTGACAGATCTTCAAACACTAGTTTAAGCAAAGGACTATTCATCCGTTTATATTAGCAACAGTAGGTACTAGCACCACTAACAACAACAACAAAGCACTTCTATTTATTTATCGAATTCATAAAGCTTCCGCCGCC

**ATGGCTTCTAACTTTACTCAGTTCGTTCTCGTCGACAATGGCGGAACTGGCGACGTGACTGTCGCCCCAAGCAACTTCGCTAACGGGATCGCTGAATGGATCAGCTCTAACTCGCGTTCACAGGCTTACAAAGTAACCTGTAGCGTTCGTCAGAGCTCTGCGCAGAATCGCAAATACACCATCAAAGTCGAGGTGCCTAAAGGCGCCTGGCGTTCGTACTTAAATATGGAACTAACCATTCCAATTTTCGCCACGAATTCCGACTGCGAGCTTATTGTTAAGGCAATGCAAGGTCTCCTAAAAGATGGAAACCCGATTCCCTCAGCAATCGCAGCAAACTCCGGCATCTACggatcaggttctcaCATGGTGAGCAAGGGCGAGGAGCTGTTCACCGGGGTGGTGCCCATCCTGGTCGAGCTGGACGGCGACGTAAACGGCCACAAGTTCAGCGTGTCCGGCGAGGGCGAGGGCGATGCCACCTACGGCAAGCTGACCCTGAAGTTCATCTGCACCACCGGCAAGCTGCCCGTGCCCTGGCCCACCCTCGTGACCACCTTCGGCTACGGCCTGCAGTGCTTCGCCCGCTACCCCGACCACATGAAGCAGCACGACTTCTTCAAGTCCGCCATGCCCGAAGGCTACGTCCAGGAGCGCACCATCTTCTTCAAGGACGACGGCAACTACAAGACCCGCGCCGAGGTGAAGTTCGAGGGCGACACCCTGGTGAACCGCATCGAGCTGAAGGGCATCGACTTCAAGGAGGACGGCAACATCCTGGGGCACAAGCTGGAGTACAACTACAACAGCCACAACGTCTATATCATGGCCGACAAGCAGAAGAACGGCATCAAGGTGAACTTCAAGATCCGCCACAACATCGAGGACGGCAGCGTGCAGCTCGCCGACCACTACCAGCAGAACACCCCCATCGGCGACGGCCCCGTGCTGCTGCCCGACAACCACTACCTGAGCTACCAGTCCGCCCTGAGCAAAGACCCCAACGAGAAGCGCGATCACATGGTCCTGCTGGAGTTCGTGACCGCCGCCGGGATCACTCTCGGCATGGACGAGCTGTACAAGGGTGGACGCGGAGGTCGTGGAGGTCGTGGTCACAAACGTTCACGCGAGTAA**

GgatcccaccgggttgtgtggccaaatttgttctgtagttgctgtgagttgacacggctagtgcttatgattttcctcgcgcgtggtgcctgtactcagccctatgccttatatgcaacacatttacgtacagcgcacaagaggagagaagatcacttgaagataataaatatagggttgtaggcatcttgtttaactcaaattttctcgccttggtgtgtcgacatgattgaaatagtgccaccagttgtgtttgatgcgtttgttatctatgcagcattgcacagcaaggtcttctgaaattcatgttttttttttttactctgcattgcagtctccgctcttatttagttttgctttacgtaaggtctcgttactgccataaaatacaTATG

**p3605 alpha tubulin 5’- MS2bp-eGFP-nls ORF – actin 3’**

TTAATTAAtagaaagtgtgacaacgtcgcaccatgtgtaggttttcatttatgttctttctttcttttttttgtgaatttgttttctgtctcaaatgtttttaattcgcttgggacctatgtttttcttgtttttttgctcaccctttgtgtaggaggcaccctgtcacgtctgtggttgcgtgtatgccttccttccccttattcgcttcttcctgtcgtgtcacacctctttctctctctccctttccgccttttctttcaatcttgttttctcgaccagccctactagaggagaaagaatagtaaccctttcatcaaagaaaatagttcaaacgaattCATaAAGCTTCCGCCGCC

**ATGGCTTCTAACTTTACTCAGTTCGTTCTCGTCGACAATGGCGGAACTGGCGACGTGACTGTCGCCCCAAGCAACTTCGCTAACGGGATCGCTGAATGGATCAGCTCTAACTCGCGTTCACAGGCTTACAAAGTAACCTGTAGCGTTCGTCAGAGCTCTGCGCAGAATCGCAAATACACCATCAAAGTCGAGGTGCCTAAAGGCGCCTGGCGTTCGTACTTAAATATGGAACTAACCATTCCAATTTTCGCCACGAATTCCGACTGCGAGCTTATTGTTAAGGCAATGCAAGGTCTCCTAAAAGATGGAAACCCGATTCCCTCAGCAATCGCAGCAAACTCCGGCATCTACggatcaggttctcaCATGGTGAGCAAGGGCGAGGAGCTGTTCACCGGGGTGGTGCCCATCCTGGTCGAGCTGGACGGCGACGTAAACGGCCACAAGTTCAGCGTGTCCGGCGAGGGCGAGGGCGATGCCACCTACGGCAAGCTGACCCTGAAGTTCATCTGCACCACCGGCAAGCTGCCCGTGCCCTGGCCCACCCTCGTGACCACCCTGACCTACGGCGTGCAGTGCTTCAGCCGCTACCCCGACCACATGAAGCAGCACGACTTCTTCAAGTCCGCCATGCCCGAAGGCTACGTCCAGGAGCGCACCATCTTCTTCAAGGACGACGGCAACTACAAGACCCGCGCCGAGGTGAAGTTCGAGGGcGACACCCTGGTGAACCGCATCGAGCTGAAGGGCATCGACTTCAAGGAGGACGGCAACATCCTGGGGCACAAGCTGGAGTACAACTACAACAGCCACAACGTCTATATCATGGCCGACAAGCAGAAGAACGGCATCAAGGTGAACTTCAAGATCCGCCACAACATCGAGGACGGCAGCGTGCAGCTCGCCGACCACTACCAGCAGAACACCCCCATCGGcGACGGCCCCGTGCTGCTGCCCGACAACCACTACCTGAGCACCCAGTCCGCCCTGAGCAAAGACCCCAACGAGAAGCGCGATCACATGGTCCTGCTGGAGTTCGTGACCGCCGCCGGGATCACTCTCGGCATGGACGAGCTGTACAAGGGTGGACGCGGAGGTCGTGGAGGTCGTGGTCACAAACGTTCACGCGAGTAA**

GgatcccaccgggttgtgtggccaaatttgttctgtagttgctgtgagttgacacggctagtgcttatgattttcctcgcgcgtggtgcctgtactcagccctatgccttatatgcaacacatttacgtacagcgcacaagaggagagaagatcacttgaagataataaatatagggttgtaggcatcttgtttaactcaaattttctcgccttggtgtgtcgacatgattgaaatagtgccaccagttgtgtttgatgcgtttgttatctatgcagcattgcacagcaaggtcttctgaaattcatgttttttttttttactctgcattgcagtctccgctcttatttagttttgctttacgtaaggtctcgttactgccataaaatacaTATG

**p3998 alpha tubulin 5’- MS2bp (altered codons)-eGFP-nls ORF – actin 3’**

TTAATTAAtagaaagtgtgacaacgtcgcaccatgtgtaggttttcatttatgttctttctttcttttttttgtgaatttgttttctgtctcaaatgtttttaattcgcttgggacctatgtttttcttgtttttttgctcaccctttgtgtaggaggcaccctgtcacgtctgtggttgcgtgtatgccttccttccccttattcgcttcttcctgtcgtgtcacacctctttctctctctccctttccgccttttctttcaatcttgttttctcgaccagccctactagaggagaaagaatagtaaccctttcatcaaagaaaatagttcaaacgaattCATaAAGCTTCCGCCGCC

**ATGtcggcctcgaatttcacccaattcgtcctagtcgacaatggggggaccggggacgtcaccgtcgccccttcgaatttcgccaatgggatagccgaatggatatcgtcgaattcgagatcgcaagcctataaagtcacctgttcggtcagacaatcgtcggcccaaaatagaaaatataccataaaagtcgaagtccctaaaggggcctggagatcgtatctaaatatggaactaaccatacctatattcgccaccaattcggactgtgaactaatagtcaaagccatgcaagggctactaaaagacgggaatcctataccttcggccatagccgccaattcggggatatatgggtcggggtcgcatatggtctcgaaaggggaagaactattcaccggggtcgtccctatactagtcgaactagacggggacgtcaatgggcataaattctcggtctcgggggaaggggaaggggacgccacctatgggaaactaaccctaaaattcatatgtaccaccgggaaactacctgtcccttggcctaccctagtcaccaccttcgggtatgggctacaatgtttcgccagatatcctgaccatatgaaacaacatgacttcttcaaatcggccatgcctgaagggtatgtccaagaaagaaccatattcttcaaagacgacgggaattataaaaccagagccgaagtcaaattcgaaggggacaccctagtcaatagaatagaactaaaagggatagacttcaaagaagacgggaatatactagggcataaactagaatataattataattcgcataatgtctatataatggccgacaaacaaaaaaatgggataaaagtcaatttcaaaataagacataatatagaagacgggtcggtccaactagccgaccattatcaacaaaatacccctataggggacgggcctgtcctactacctgacaatcattatctatcgtatcaatcggccctatcgaaagaccctaatgaaaaaagagaccatatggtcctactagaattcgtcaccgccgccgggataaccctagggatggacgaactatataaaggggggagagggggcagaggcggaagaggacataaaagatcgagagaaTAA**

GgatcccaccgggttgtgtggccaaatttgttctgtagttgctgtgagttgacacggctagtgcttatgattttcctcgcgcgtggtgcctgtactcagccctatgccttatatgcaacacatttacgtacagcgcacaagaggagagaagatcacttgaagataataaatatagggttgtaggcatcttgtttaactcaaattttctcgccttggtgtgtcgacatgattgaaatagtgccaccagttgtgtttgatgcgtttgttatctatgcagcattgcacagcaaggtcttctgaaattcatgttttttttttttactctgcattgcagtctccgctcttatttagttttgctttacgtaaggtctcgttactgccataaaatacaTATG

**p3854 synthetic 5’- MS2bp-eGFP-nls ORF – actin 3’**

TTAATTAATAGACGCGGACGGGGCATTTCCCGTTCGTCATTAGCAGTAGGTAATGAAGATGTTTGTTTCTCGTCCCCTTTCTCCTTCGTCCTTCTGTCATTTTGTTCTTTTGTGTTTATGTTTTGTTGTTGTTTTCTTTAATTTTTTTTTTTCTTCCACGTTTGTGTACATCCGCGCGCCACTCTATTCAGAGAGCCACGGATAGTAGAGGAGGTGGGAAGGGTATATGAGGGACACGCGTACCATGATGTGGGATGTATTGGGGTCCCTGTCTGTCCTTACGTGACTATGTATGAACCGTCACGTGTAAGATGAGCTAGTGAGATCAACAGTACAACTCATCAACACGCCTTCTTCTCGTTAAATGTACACAATCTTGATCCTCCACCTTTATGGGTCCCATTGTTTGCCTCTTCCGCTGTGTGGAGTGCGCCTACACGCACTTCTCACTTCGTAAGTGGTGGTGGCGTAAGTATTGCCTAATGTTGACTCTATATTCTCCTCTCCTCACCCCCTCGCGGTGCTGATTTCTGACAGATCTTCAAACACTAGTAAAATAAAATTCACACTTGGAATTCCTTTGTGTTACATTCTTGATCGCTCGCTGACATTCATTCCAAAGCTTCCGCCGCC

**ATGGCTTCTAACTTTACTCAGTTCGTTCTCGTCGACAATGGCGGAACTGGCGACGTGACTGTCGCCCCAAGCAACTTCGCTAACGGGATCGCTGAATGGATCAGCTCTAACTCGCGTTCACAGGCTTACAAAGTAACCTGTAGCGTTCGTCAGAGCTCTGCGCAGAATCGCAAATACACCATCAAAGTCGAGGTGCCTAAAGGCGCCTGGCGTTCGTACTTAAATATGGAACTAACCATTCCAATTTTCGCCACGAATTCCGACTGCGAGCTTATTGTTAAGGCAATGCAAGGTCTCCTAAAAGATGGAAACCCGATTCCCTCAGCAATCGCAGCAAACTCCGGCATCTACggatcaggttctcaCATGGTGAGCAAGGGCGAGGAGCTGTTCACCGGGGTGGTGCCCATCCTGGTCGAGCTGGACGGCGACGTAAACGGCCACAAGTTCAGCGTGTCCGGCGAGGGCGAGGGCGATGCCACCTACGGCAAGCTGACCCTGAAGTTCATCTGCACCACCGGCAAGCTGCCCGTGCCCTGGCCCACCCTCGTGACCACCTTCGGCTACGGCCTGCAGTGCTTCGCCCGCTACCCCGACCACATGAAGCAGCACGACTTCTTCAAGTCCGCCATGCCCGAAGGCTACGTCCAGGAGCGCACCATCTTCTTCAAGGACGACGGCAACTACAAGACCCGCGCCGAGGTGAAGTTCGAGGGCGACACCCTGGTGAACCGCATCGAGCTGAAGGGCATCGACTTCAAGGAGGACGGCAACATCCTGGGGCACAAGCTGGAGTACAACTACAACAGCCACAACGTCTATATCATGGCCGACAAGCAGAAGAACGGCATCAAGGTGAACTTCAAGATCCGCCACAACATCGAGGACGGCAGCGTGCAGCTCGCCGACCACTACCAGCAGAACACCCCCATCGGCGACGGCCCCGTGCTGCTGCCCGACAACCACTACCTGAGCTACCAGTCCGCCCTGAGCAAAGACCCCAACGAGAAGCGCGATCACATGGTCCTGCTGGAGTTCGTGACCGCCGCCGGGATCACTCTCGGCATGGACGAGCTGTACAAGGGTGGACGCGGAGGTCGTGGAGGTCGTGGTCACAAACGTTCACGCGAGTAA**

GgatcccaccgggttgtgtggccaaatttgttctgtagttgctgtgagttgacacggctagtgcttatgattttcctcgcgcgtggtgcctgtactcagccctatgccttatatgcaacacatttacgtacagcgcacaagaggagagaagatcacttgaagataataaatatagggttgtaggcatcttgtttaactcaaattttctcgccttggtgtgtcgacatgattgaaatagtgccaccagttgtgtttgatgcgtttgttatctatgcagcattgcacagcaaggtcttctgaaattcatgttttttttttttactctgcattgcagtctccgctcttatttagttttgctttacgtaaggtctcgttactgccataaaatacaTATG

**p3866 synthetic 5’plus splice site AG->AA mutation - MS2bp-eGFP-nls ORF – actin 3’**

TTAATTAATAGACGCGGACGGGGCATTTCCCGTTCGTCATTAGCAGTAGGTAATGAAGATGTTTGTTTCTCGTCCCCTTTCTCCTTCGTCCTTCTGTCATTTTGTTCTTTTGTGTTTATGTTTTGTTGTTGTTTTCTTTAATTTTTTTTTTTCTTCCACGTTTGTGTACATCCGCGCGCCACTCTATTCAGAGAGCCACGGATAGTAGAGGAGGTGGGAAGGGTATATGAGGGACACGCGTACCATGATGTGGGATGTATTGGGGTCCCTGTCTGTCCTTACGTGACTATGTATGAACCGTCACGTGTAAGATGAGCTAGTGAGATCAACAGTACAACTCATCAACACGCCTTCTTCTCGTTAAATGTACACAATCTTGATCCTCCACCTTTATGGGTCCCATTGTTTGCCTCTTCCGCTGTGTGGAGTGCGCCTACACGCACTTCTCACTTCGTAAGTGGTGGTGGCGTAAGTATTGCCTAATGTTGACTCTATATTCTCCTCTCCTCACCCCCTCGCGGTGCTGATTTCTGACAGATCTTCAAACACTAaTAAAATAAAATTCACACTTGGAATTCCTTTGTGTTACATTCTTGATCGCTCGCTGACATTCATTCCAAAGCTTCCGCCGCC

**ATGGCTTCTAACTTTACTCAGTTCGTTCTCGTCGACAATGGCGGAACTGGCGACGTGACTGTCGCCCCAAGCAACTTCGCTAACGGGATCGCTGAATGGATCAGCTCTAACTCGCGTTCACAGGCTTACAAAGTAACCTGTAGCGTTCGTCAGAGCTCTGCGCAGAATCGCAAATACACCATCAAAGTCGAGGTGCCTAAAGGCGCCTGGCGTTCGTACTTAAATATGGAACTAACCATTCCAATTTTCGCCACGAATTCCGACTGCGAGCTTATTGTTAAGGCAATGCAAGGTCTCCTAAAAGATGGAAACCCGATTCCCTCAGCAATCGCAGCAAACTCCGGCATCTACggatcaggttctcaCATGGTGAGCAAGGGCGAGGAGCTGTTCACCGGGGTGGTGCCCATCCTGGTCGAGCTGGACGGCGACGTAAACGGCCACAAGTTCAGCGTGTCCGGCGAGGGCGAGGGCGATGCCACCTACGGCAAGCTGACCCTGAAGTTCATCTGCACCACCGGCAAGCTGCCCGTGCCCTGGCCCACCCTCGTGACCACCTTCGGCTACGGCCTGCAGTGCTTCGCCCGCTACCCCGACCACATGAAGCAGCACGACTTCTTCAAGTCCGCCATGCCCGAAGGCTACGTCCAGGAGCGCACCATCTTCTTCAAGGACGACGGCAACTACAAGACCCGCGCCGAGGTGAAGTTCGAGGGCGACACCCTGGTGAACCGCATCGAGCTGAAGGGCATCGACTTCAAGGAGGACGGCAACATCCTGGGGCACAAGCTGGAGTACAACTACAACAGCCACAACGTCTATATCATGGCCGACAAGCAGAAGAACGGCATCAAGGTGAACTTCAAGATCCGCCACAACATCGAGGACGGCAGCGTGCAGCTCGCCGACCACTACCAGCAGAACACCCCCATCGGCGACGGCCCCGTGCTGCTGCCCGACAACCACTACCTGAGCTACCAGTCCGCCCTGAGCAAAGACCCCAACGAGAAGCGCGATCACATGGTCCTGCTGGAGTTCGTGACCGCCGCCGGGATCACTCTCGGCATGGACGAGCTGTACAAGGGTGGACGCGGAGGTCGTGGAGGTCGTGGTCACAAACGTTCACGCGAGTAA**

GgatcccaccgggttgtgtggccaaatttgttctgtagttgctgtgagttgacacggctagtgcttatgattttcctcgcgcgtggtgcctgtactcagccctatgccttatatgcaacacatttacgtacagcgcacaagaggagagaagatcacttgaagataataaatatagggttgtaggcatcttgtttaactcaaattttctcgccttggtgtgtcgacatgattgaaatagtgccaccagttgtgtttgatgcgtttgttatctatgcagcattgcacagcaaggtcttctgaaattcatgttttttttttttactctgcattgcagtctccgctcttatttagttttgctttacgtaaggtctcgttactgccataaaatacaTATG

**p3943 synthetic 5’plus splice site AG->AA mutation plus altered sequence around initiation codon - MS2bp-eGFP-nls ORF – actin 3’**

TTAATTAATAGACGCGGACGGGGCATTTCCCGTTCGTCATTAGCAGTAGGTAATGAAGATGTTTGTTTCTCGTCCCCTTTCTCCTTCGTCCTTCTGTCATTTTGTTCTTTTGTGTTTATGTTTTGTTGTTGTTTTCTTTAATTTTTTTTTTTCTTCCACGTTTGTGTACATCCGCGCGCCACTCTATTCAGAGAGCCACGGATAGTAGAGGAGGTGGGAAGGGTATATGAGGGACACGCGTACCATGATGTGGGATGTATTGGGGTCCCTGTCTGTCCTTACGTGACTATGTATGAACCGTCACGTGTAAGATGAGCTAGTGAGATCAACAGTACAACTCATCAACACGCCTTCTTCTCGTTAAATGTACACAATCTTGATCCTCCACCTTTATGGGTCCCATTGTTTGCCTCTTCCGCTGTGTGGAGTGCGCCTACACGCACTTCTCACTTCGTAAGTGGTGGTGGCGTAAGTATTGCCTAATGTTGACTCTATATTCTCCTCTCCTCACCCCCTCGCGGTGCTGATTTCTGACAGATCTTCAAACACTAaTAAAATAAAATTCACACTTGGAATTCCTTTGTGTTACATTCTTGATCGCTCGCTGACATTCATTCCAAAGCTTttttt

**ATGtctGCTTCTAACTTTACTCAGTTCGTTCTCGTCGACAATGGCGGAACTGGCGACGTGACTGTCGCCCCAAGCAACTTCGCTAACGGGATCGCTGAATGGATCAGCTCTAACTCGCGTTCACAGGCTTACAAAGTAACCTGTAGCGTTCGTCAGAGCTCTGCGCAGAATCGCAAATACACCATCAAAGTCGAGGTGCCTAAAGGCGCCTGGCGTTCGTACTTAAATATGGAACTAACCATTCCAATTTTCGCCACGAATTCCGACTGCGAGCTTATTGTTAAGGCAATGCAAGGTCTCCTAAAAGATGGAAACCCGATTCCCTCAGCAATCGCAGCAAACTCCGGCATCTACggatcaggttctcaCATGGTGAGCAAGGGCGAGGAGCTGTTCACCGGGGTGGTGCCCATCCTGGTCGAGCTGGACGGCGACGTAAACGGCCACAAGTTCAGCGTGTCCGGCGAGGGCGAGGGCGATGCCACCTACGGCAAGCTGACCCTGAAGTTCATCTGCACCACCGGCAAGCTGCCCGTGCCCTGGCCCACCCTCGTGACCACCTTCGGCTACGGCCTGCAGTGCTTCGCCCGCTACCCCGACCACATGAAGCAGCACGACTTCTTCAAGTCCGCCATGCCCGAAGGCTACGTCCAGGAGCGCACCATCTTCTTCAAGGACGACGGCAACTACAAGACCCGCGCCGAGGTGAAGTTCGAGGGCGACACCCTGGTGAACCGCATCGAGCTGAAGGGCATCGACTTCAAGGAGGACGGCAACATCCTGGGGCACAAGCTGGAGTACAACTACAACAGCCACAACGTCTATATCATGGCCGACAAGCAGAAGAACGGCATCAAGGTGAACTTCAAGATCCGCCACAACATCGAGGACGGCAGCGTGCAGCTCGCCGACCACTACCAGCAGAACACCCCCATCGGCGACGGCCCCGTGCTGCTGCCCGACAACCACTACCTGAGCTACCAGTCCGCCCTGAGCAAAGACCCCAACGAGAAGCGCGATCACATGGTCCTGCTGGAGTTCGTGACCGCCGCCGGGATCACTCTCGGCATGGACGAGCTGTACAAGGGTGGACGCGGAGGTCGTGGAGGTCGTGGTCACAAACGTTCACGCGAGTAA**

GgatcccaccgggttgtgtggccaaatttgttctgtagttgctgtgagttgacacggctagtgcttatgattttcctcgcgcgtggtgcctgtactcagccctatgccttatatgcaacacatttacgtacagcgcacaagaggagagaagatcacttgaagataataaatatagggttgtaggcatcttgtttaactcaaattttctcgccttggtgtgtcgacatgattgaaatagtgccaccagttgtgtttgatgcgtttgttatctatgcagcattgcacagcaaggtcttctgaaattcatgttttttttttttactctgcattgcagtctccgctcttatttagttttgctttacgtaaggtctcgttactgccataaaatacaTATG

**p3965 synthetic 5’ plus splice site AG->AA mutation plus altered sequence around initiation codon - MS2bp-eGFP-nls ORF – RAB28 3’**

TTAATTAATAGACGCGGACGGGGCATTTCCCGTTCGTCATTAGCAGTAGGTAATGAAGATGTTTGTTTCTCGTCCCCTTTCTCCTTCGTCCTTCTGTCATTTTGTTCTTTTGTGTTTATGTTTTGTTGTTGTTTTCTTTAATTTTTTTTTTTCTTCCACGTTTGTGTACATCCGCGCGCCACTCTATTCAGAGAGCCACGGATAGTAGAGGAGGTGGGAAGGGTATATGAGGGACACGCGTACCATGATGTGGGATGTATTGGGGTCCCTGTCTGTCCTTACGTGACTATGTATGAACCGTCACGTGTAAGATGAGCTAGTGAGATCAACAGTACAACTCATCAACACGCCTTCTTCTCGTTAAATGTACACAATCTTGATCCTCCACCTTTATGGGTCCCATTGTTTGCCTCTTCCGCTGTGTGGAGTGCGCCTACACGCACTTCTCACTTCGTAAGTGGTGGTGGCGTAAGTATTGCCTAATGTTGACTCTATATTCTCCTCTCCTCACCCCCTCGCGGTGCTGATTTCTGACAGATCTTCAAACACTAaTAAAATAAAATTCACACTTGGAATTCCTTTGTGTTACATTCTTGATCGCTCGCTGACATTCATTCCAAAGCTTttttt

**ATGtctGCTTCTAACTTTACTCAGTTCGTTCTCGTCGACAATGGCGGAACTGGCGACGTGACTGTCGCCCCAAGCAACTTCGCTAACGGGATCGCTGAATGGATCAGCTCTAACTCGCGTTCACAGGCTTACAAAGTAACCTGTAGCGTTCGTCAGAGCTCTGCGCAGAATCGCAAATACACCATCAAAGTCGAGGTGCCTAAAGGCGCCTGGCGTTCGTACTTAAATATGGAACTAACCATTCCAATTTTCGCCACGAATTCCGACTGCGAGCTTATTGTTAAGGCAATGCAAGGTCTCCTAAAAGATGGAAACCCGATTCCCTCAGCAATCGCAGCAAACTCCGGCATCTACggatcaggttctcaCATGGTGAGCAAGGGCGAGGAGCTGTTCACCGGGGTGGTGCCCATCCTGGTCGAGCTGGACGGCGACGTAAACGGCCACAAGTTCAGCGTGTCCGGCGAGGGCGAGGGCGATGCCACCTACGGCAAGCTGACCCTGAAGTTCATCTGCACCACCGGCAAGCTGCCCGTGCCCTGGCCCACCCTCGTGACCACCTTCGGCTACGGCCTGCAGTGCTTCGCCCGCTACCCCGACCACATGAAGCAGCACGACTTCTTCAAGTCCGCCATGCCCGAAGGCTACGTCCAGGAGCGCACCATCTTCTTCAAGGACGACGGCAACTACAAGACCCGCGCCGAGGTGAAGTTCGAGGGCGACACCCTGGTGAACCGCATCGAGCTGAAGGGCATCGACTTCAAGGAGGACGGCAACATCCTGGGGCACAAGCTGGAGTACAACTACAACAGCCACAACGTCTATATCATGGCCGACAAGCAGAAGAACGGCATCAAGGTGAACTTCAAGATCCGCCACAACATCGAGGACGGCAGCGTGCAGCTCGCCGACCACTACCAGCAGAACACCCCCATCGGCGACGGCCCCGTGCTGCTGCCCGACAACCACTACCTGAGCTACCAGTCCGCCCTGAGCAAAGACCCCAACGAGAAGCGCGATCACATGGTCCTGCTGGAGTTCGTGACCGCCGCCGGGATCACTCTCGGCATGGACGAGCTGTACAAGGGTGGACGCGGAGGTCGTGGAGGTCGTGGTCACAAACGTTCACGCGAGTAA**

GGATCCtggtagcgacaagaaaatttccgcctttacctgtttgttgtctttttaaaatattcttactcattgtttgcgttcacacatgagaataactaccttttattattgttgttattgttcctacccaccaatgtcacgcagtaggtgttttatgcgggcttgttccccttttgcttctttccccctgcttctgttccttcttcgcaccgtatcctctttccattttttcctctactttactggcgacgtggagtatgattggaagaggtaaacttcatggattcgaattttgttccgtatggaatgcgcgtcgactccctctgttagcaaacttgggtgtgacgcgtatgtcccagaatgtttttctatttatgctacggcaatggggaatatatcttctttttaaaatatcttatattcctttgtgacgtgtatggtacacatatattttttaataggtttatatacttatttttgtttctgaagaattgctccattcgttcgttgtaacactgatctgttgttggtttatatatctttggattttattaatatgttgttttcgtttactgtcctgacttacattgtttgcactcggtgcgctagtaaagaagaattacaggtagaggttatcgtagttgttgggagatttttttttttttgacttagtagggattcgtatttcatataaccatCATATG
