# Supplementary material for: Codon choice directs constitutive mRNA levels in trypanosomes
Source: eLife. 2018 Mar 15;7:e32467. doi: 10.7554/eLife.32467 (PMC5896880; doi:10.7554/eLife.32467)
Supplement: Supplementary file 2. — (A) GFP transgene sequence after integration. 5’ region from an alpha-tubulin gene, trans-splice acceptor sites in red (Kolev et al., 2010), GFP open reading frame in green, and 3’ region from an actin gene, polyadenylation sites in blue (Kolev et al., 2010). Grey sequences added to construct for cloning of GFP variants (B) 5’UTR variants. Sequences adding secondary structure to the 5’UTR are shown in blue; the two characterised trans-splicing site AG acceptor sites are shown in red; the initiation codon is in green and other additional sequences are in grey. [file elife-32467-supp2.docx]

**A.**

GTAAGTATTGCCTAATGTTGACTCTATATTCTCCTCTCCTCACCCCCTCGCGGTGCTGATTTCTGACAGATCTTCAAACACTAGTTTAAGCAAAGGACTATTCATCCGTTTATATTAGCAACAGTAGGTACTAGCACCACTAACAACAACAACAAAGCACTTCTATTTATTTATCATAAAGCTTCAACAATGGTGAGCAAGGGCGAGGAGCTGTTCACCGGGGTGGTGCCCATCCTGGTCGAGCTGGACGGCGACGTAAACGGCCACAAGTTCAGCGTGTCCGGCGAGGGCGAGGGCGATGCCACCTACGGCAAGCTGACCCTGAAGTTCATCTGCACCACCGGCAAGCTGCCCGTGCCCTGGCCCACCCTCGTGACCACCCTGACCTACGGCGTGCAGTGCTTCAGCCGCTACCCCGACCACATGAAGCAGCACGACTTCTTCAAGTCCGCCATGCCCGAAGGCTACGTCCAGGAGCGCACCATCTTCTTCAAGGACGACGGCAACTACAAGACCCGCGCCGAGGTGAAGTTCGAGGGcGACACCCTGGTGAACCGCATCGAGCTGAAGGGCATCGACTTCAAGGAGGACGGCAACATCCTGGGGCACAAGCTGGAGTACAACTACAACAGCCACAACGTCTATATCATGGCCGACAAGCAGAAGAACGGCATCAAGGTGAACTTCAAGATCCGCCACAACATCGAGGACGGCAGCGTGCAGCTCGCCGACCACTACCAGCAGAACACCCCCATCGGCGACGGCCCCGTGCTGCTGCCCGACAACCACTACCTGAGCACCCAGTCCGCCCTGAGCAAAGACCCCAACGAGAAGCGCGATCACATGGTCCTGCTGGAGTTCGTGACCGCCGCCGGGATCACTCTCGGCATGGACGAGCTGTACAAGTAAGGATCCaccgggttgtgtggccaaatttgttctgtagttgctgtgagttgacacggctagtgcttatgattttcctcgcgtgtggtgcctgtactcagccctatgccttatttgcaacacatttacgtacagcgcacaagagaagagaagatcacttgaagataataaatatagggttgtaggcatcttgtttaactcaaattttctcgtcttggtgtgtcgacatcattgaaatagtgccaccagttgtgtttgatgcgtttgttatctatgcagtattgcacagcaaggtcttctgaaattcatgttttttttttttttttactctgcattgcagtctccgct

**B.**

5’ UTR containing 24 bp (22 GC + 2 AT) hairpin in blue (Figure 5)

AGATCTTCAAACACTAGTTTAAGCAAAGGACTATTCATCCGGGCGGCGGCGGCGGCGGCGGATATCCGCCGCCGCCGCCGCCGCCCGTTTATATTAGCAACAGTAGGTACTAGCACCACTAACAACAACAACAAAGCACTTCTATTTATTTATCGAATTCATAAAGCTTCAACAATG

5’ UTR containing EcoRV site in blue (p4669)

AGATCTTCAAACACTAGTTTAAGCAAAGGACTATTCATCCGATATCATATTAGCAACAGTAGGTACTAGCACCACTAACAACAACAACAAAGCACTTCTATTTATTTATCGAATTCATAAAGCTTCAACAATG

5’ UTR containing 18 bp (16GC + 2 AT) hairpin in blue (p4841)

AGATCTTCAAACACTAGTTTAAGCAAAGGACTATTCATCCGATTCCGCCGCCGCCGCCCATATGGGCGGCGGCGGCGGATCATATTAGCAACAGTAGGTACTAGCACCACTAACAACAACAACAAAGCACTTCTATTTATTTATCGAATTCATAAAGCTTCAACAATG

5’ UTR containing 12 bp (9GC + 3 AT) hairpin in blue (p4842)

AGATCTTCAAACACTAGTTTAAGCAAAGGACTATTCATCCGATTCCGCCGCCGCCCATATCCGGCGGCGGAATCATATTAGCAACAGTAGGTACTAGCACCACTAACAACAACAACAAAGCACTTCTATTTATTTATCGAATTCATAAAGCTTCAACAATG
