# Supplementary material for: Codon choice directs constitutive mRNA levels in trypanosomes
Source: eLife. 2018 Mar 15;7:e32467. doi: 10.7554/eLife.32467 (PMC5896880; doi:10.7554/eLife.32467)
Supplement: Supplementary file 3. [file elife-32467-supp3.docx]

>eGFP

ATGGTGAGCAAGGGCGAGGAGCTGTTCACCGGGGTGGTGCCCATCCTGGTCGAGCTGGACGGCGACGTAAACGGCCACAAGTTCAGCGTGTCCGGCGAGGGCGAGGGCGATGCCACCTACGGCAAGCTGACCCTGAAGTTCATCTGCACCACCGGCAAGCTGCCCGTGCCCTGGCCCACCCTCGTGACCACCCTGACCTACGGCGTGCAGTGCTTCAGCCGCTACCCCGACCACATGAAGCAGCACGACTTCTTCAAGTCCGCCATGCCCGAAGGCTACGTCCAGGAGCGCACCATCTTCTTCAAGGACGACGGCAACTACAAGACCCGCGCCGAGGTGAAGTTCGAGGGcGACACCCTGGTGAACCGCATCGAGCTGAAGGGCATCGACTTCAAGGAGGACGGCAACATCCTGGGGCACAAGCTGGAGTACAACTACAACAGCCACAACGTCTATATCATGGCCGACAAGCAGAAGAACGGCATCAAGGTGAACTTCAAGATCCGCCACAACATCGAGGACGGCAGCGTGCAGCTCGCCGACCACTACCAGCAGAACACCCCCATCGGcGACGGCCCCGTGCTGCTGCCCGACAACCACTACCTGAGCACCCAGTCCGCCCTGAGCAAAGACCCCAACGAGAAGCGCGATCACATGGTCCTGCTGGAGTTCGTGACCGCCGCCGGGATCACTCTCGGCATGGACGAGCTGTACAAGTAA

>GFP_71

atggtgagtaagggtgaggagctttttaccggtgtggttcctattcttgtggagttagatggggatgtgaatggtcataagtttagcgtttctggtgaaggtgaaggcgatgccacttacggaaaattaacccttaagtttatatgcactacgggtaagctccctgtgccttggccgacccttgtgactaccctcacttacggtgtacagtgcttctctagataccccgaccacatgaagcagcacgacttcttcaaaagcgccatgcccgaaggctacgtgcaggagaggacgatcttcttcaaggacgacgggaactataagacccgagcagaggtgaaattcgagggggacaccctagtaaacaggatcgagttgaaggggatcgacttcaaggaggacgggaatatcctggggcacaagttggagtacaactacaactcccacaacgtgtacataatggcagacaaacaaaagaacggaatcaaagttaacttcaagatcaggcacaatatcgaggatggcagcgttcagcttgcagatcattaccaacagaacacaccgatcggcgacggcccggtactgttgccggataaccactacctgtcgacgcagtctgccctatcgaaagaccctaacgaaaaaagagaccatatggtgctattagagtttgtgactgcagcaggtattacacttggtatggacgaactttataaataa

>GFP_183

atggtcagtaaaggcgaagaactgttcaccggtgttgtaccaatcctagttgagttggatggggatgtgaacggtcacaaattcagcgtttctggtgaaggtgaaggcgacgcaacctacggcaaattgacgctgaaattcatctgtaccactggcaaactgccggtaccgtggccgactctggtaaccactctgacttacggagtacagtgtttctctagatacccggatcacatgaaacagcacgatttcttcaaaagcgcgatgccagaaggttacgtacaagaaagaaccatcttcttcaaagatgatggaaactacaaaacacgcgcagaggtcaaatttgaaggcgacaccctggtaaacagaatcgaattaaaaggcatcgatttcaaagaagatggaaacatcctaggccacaaattggaatacaactacaactctcacaacgtttacatcatggctgataaacagaaaaacggcataaaagttaacttcaaaatcagacacaacatcgaagacggtagcgtacagctggccgatcactatcagcagaacaccccgatcggtgatggtccggtactgttgccggataaccactacctgtccactcagtctgccctctccaaagacccgaacgaaaaaagagatcacatggttctgctggagttcgtaaccgctgcgggcatcaccctgggcatggacgaactgtacaaataa

>GFP_188

atggtcagtaaaggcgaagaactgttcaccggtgttgtaccaatcctagttgagttggatggggatgtgaacggtcacaaattcagcgtttctggtgaaggtgaaggcgacgcaacctacggcaaattgacgctgaaattcatctgtaccactggcaaactgccggtaccgtggccgactctggtaaccactctgacttacggagtacagtgtttctctagatacccggaccacatgaaacagcacgacttcttcaagagcgctatgcccgaaggctacgttcaggaaagaactatcttcttcaaagacgatggcaactacaaaacacgcgccgaggtaaaattcgaaggcgacaccctggtaaacagaatcgaattaaaaggcatcgatttcaaagaagacggaaatattcttggccacaagttagaatacaactacaactctcacaacgtttacatcatggcagacaaacagaaaaacggcatcaaagttaacttcaaaatcagacacaacatcgaagacggtagcgtacagctggccgatcactatcagcagaacaccccgatcggtgatggtccggtactgttgccggataaccactacctgtccactcagtctgccctctccaaagacccgaacgaaaaaagagatcacatggttctgctggagttcgtaaccgctgcgggcatcaccctgggcatggacgaactgtacaaataa

>GFP_194

atggtgagcaagggggaagagctgttcactggtgtggtgcctatacttgttgagttggacggagatgtgaatggacataagtttagtgtttctggggagggtgagggtgatgctacgtacgggaagttgactctcaaatttatttgtactaccggaaagctgcctgtaccctggcccactctagtaacgacgctcacctacggcgtgcaatgcttctctagatacccagaccacatgaaacaacacgacttcttcaagagcgccatgccagagggatacgtacaggaaaggacaatattcttcaaggatgacggcaactacaaaacccgagcggaagtcaaattcgagggagacactctcgtaaacagaatcgaattgaaagggatagattttaaggaggatggtaacatacttggccataagttagaatataattataattcacacaacgtatacatcatggccgataaacagaagaacgggatcaaggttaactttaagattaggcacaacatagaggacgggagtgtccagctggcggaccactaccagcagaatacgccaataggggacggtcccgtgctgttgccagataatcattatctttccacgcaatcggcgctctcaaaggacccaaacgagaagagggatcatatggttctgttggagttcgtcacagctgcgggtatcaccctaggcatggacgaactttacaaataa

>GFP_226

atggtgagcaaaggtgaggaactttttacaggagtagtacctattcttgtagagttagatggtgatgtgaatggccataaatttagtgtttctggtgaaggagagggcgatgcgacttatggcaagttgacccttaaatttatttgtactactggtaaacttcctgttccttggcctactctcgtgacaactcttacttacggagtccaatgcttctctagatacccggaccacatgaagcagcacgatttcttcaaaagcgccatgcctgagggatacgtccaagaaaggacgatcttcttcaaggacgacgggaactacaagacacgggcggaggtgaaatttgagggggacacgctcgtgaacaggattgagttgaaggggattgacttcaaggaggacggcaatatactcggacacaaattagagtacaactacaactcgcacaacgtctacattatggccgataagcagaagaacggcattaaggttaacttcaagatcaggcacaatatcgaggatggcagcgttcagcttgcagatcattaccaacagaacacaccgatcggcgacggcccggtactgttgccggataaccactacctgtcgacgcagtctgccctatcgaaagaccctaacgaaaaaagagaccatatggtgctattagagtttgtgactgcagcaggtattacacttggtatggacgaactttataaataa

>GFP_102

atggtcagtaagggggaagaactatttacaggagtagtacctatactagtcgagttagatggggatgtgaatggccacaaatttagtgtttctggtgaaggtgaaggtgatgcaacgtatgggaaattgaccctgaagtttatatgtactactggtaaactacctgttccatggcctaccctagtcaccaccctcacatacggcgtccaatgcttctctagatatcctgatcatatgaaacaacatgatttttttaaaagtgccatgccagaaggatacgtgcaggagaggacgattttcttcaaggacgacggaaactacaagacgcgcgcggaggtcaagttcgaaggagatacactagtcaatagaatcgaattaaaaggaatcgattttaaagaagatggaaatatacttggccataaattagaatataattataattcacataatgtttatatcatggccgataagcagaagaatgggatcaaggttaactttaaaattagacataacattgaagatggaagtgttcaacttgcggaccattatcaacagaatacgccaatcggtgatggtcctgttctattaccagacaatcattacctatcaacacaatcagcgctctcgaaagaccctaatgaaaaaagagatcatatggttctattagagtttgttactgctgccggtatcaccctaggaatggatgaactatataaataa

>GFP_S1

ATGGTCTCGAAAGGGGAAGAACTATTCACCGGGGTCGTCCCTATACTAGTCGAACTAGACGGGGACGTCAATGGGCATAAATTCTCGGTCTCGGGGGAAGGGGAAGGGGACGCCACCTATGGGAAACTAACCCTAAAATTCATATGTACCACCGGGAAACTACCTGTCCCTTGGCCTACCCTAGTCACCACCCTAACCTATGGGGTCCAATGTTTCTCGAGATATCCTGACCATATGAAACAACATGACTTCTTCAAATCGGCCATGCCTGAAGGGTATGTCCAAGAAAGAACCATATTCTTCAAAGACGACGGGAATTATAAAACCAGAGCCGAAGTCAAATTCGAAGGGGACACCCTAGTCAATAGAATAGAACTAAAAGGGATAGACTTCAAAGAAGACGGGAATATACTAGGGCATAAACTAGAATATAATTATAATTCGCATAATGTCTATATAATGGCCGACAAACAAAAAAATGGGATAAAAGTCAATTTCAAAATAAGACATAATATAGAAGACGGGTCGGTCCAACTAGCCGACCATTATCAACAAAATACCCCTATAGGGGACGGGCCTGTCCTACTACCTGACAATCATTATCTATCGACCCAATCGGCCCTATCGAAAGACCCTAATGAAAAAAGAGACCATATGGTCCTACTAGAATTCGTCACCGCCGCCGGGATAACCCTAGGGATGGACGAACTATATAAATAA

>GFP_S2

ATGGTCTCGAAAGGTGAAGAACTATTCACCGGGGTCGTCCCTATACTAGTCGAACTAGACGGGGACGTCAATGGGCATAAATTCTCGGTCTCGGGGGAAGGGGAAGGGGACGCCACCTATGGGAAACTAACACTAAAATTCATATGTACCACAGGTAAGCTACCAGTCCCTTGGCCAACCCTAGTCACCACCCTAACCTATGGGGTCCAGTGCTTCTCGAGATATCCTGACCATATGAAACAACATGACTTCTTCAAGAGTGCCATGCCAGAGGGTTATGTCCAAGAACGTACCATATTCTTCAAAGACGACGGGAATTATAAAACAAGAGCCGAAGTCAAGTTCGAAGGGGACACCCTAGTCAATAGAATAGAACTAAAAGGGATAGACTTCAAAGAAGACGGGAATATACTAGGTCATAAACTAGAATATAATTATAATTCGCATAATGTCTATATAATGGCCGACAAACAAAAAAATGGGATAAAAGTCAATTTCAAAATACGTCATAATATAGAAGACGGGTCGGTGCAACTAGCCGACCATTATCAACAAAACACACCTATAGGGGACGGGCCTGTCCTACTACCTGACAATCATTACCTAAGTACCCAGTCGGCCCTATCGAAAGACCCTAATGAGAAAAGAGACCATATGGTCCTACTAGAATTCGTCACCGCCGCCGGGATTACCCTAGGGATGGATGAACTATATAAATAA

>GFP_S3

ATGGTCTCGAAAGGTGAGGAACTATTCACCGGGGTCGTCCCTATACTTGTCGAACTAGATGGTGACGTCAATGGGCATAAATTCTCGGTCTCGGGGGAAGGTGAAGGGGACGCCACATATGGGAAACTAACACTAAAATTCATATGTACCACCGGTAAGCTACCTGTCCCTTGGCCAACCCTAGTCACCACCCTAACCTACGGGGTCCAATGTTTCTCGCGTTATCCTGACCATATGAAACAACATGATTTCTTCAAAAGTGCCATGCCTGAAGGGTACGTCCAAGAAAGAACCATATTCTTCAAAGACGACGGGAATTATAAAACAAGAGCCGAAGTCAAATTCGAAGGGGATACACTAGTCAATAGAATTGAACTTAAAGGGATAGACTTCAAAGAAGACGGGAATATACTAGGGCATAAACTAGAGTACAATTACAATTCGCATAATGTCTACATAATGGCCGATAAACAAAAAAATGGTATAAAAGTCAATTTTAAAATAAGACATAACATAGAAGACGGGTCGGTCCAACTAGCCGACCACTACCAGCAAAATACCCCTATAGGGGATGGGCCTGTCCTACTACCTGATAATCATTATCTAAGTACACAAAGTGCCCTATCGAAAGATCCTAATGAGAAACGTGACCATATGGTCCTACTAGAATTCGTCACCGCAGCAGGGATAACACTAGGGATGGACGAACTATATAAGTAA

>GFP_S4

ATGGTCTCGAAAGGTGAAGAACTTTTCACAGGGGTCGTCCCTATACTAGTCGAACTAGATGGGGATGTCAATGGGCATAAATTCAGTGTCTCGGGGGAAGGGGAAGGTGACGCCACCTATGGGAAACTAACCCTAAAGTTCATATGTACCACAGGGAAACTTCCAGTGCCTTGGCCTACACTAGTCACCACCCTAACCTATGGGGTCCAATGTTTCTCGAGATATCCTGACCATATGAAACAACATGACTTTTTCAAATCGGCCATGCCAGAGGGTTACGTCCAAGAAAGAACCATATTTTTCAAGGACGACGGGAACTATAAAACCAGAGCAGAAGTCAAATTTGAAGGGGACACACTTGTCAATAGAATAGAACTAAAGGGGATAGACTTCAAAGAAGACGGGAACATACTTGGGCACAAACTTGAATATAATTATAATTCGCATAATGTCTATATAATGGCCGACAAACAAAAAAATGGGATAAAGGTGAATTTCAAAATACGTCATAACATTGAAGATGGGTCGGTCCAACTAGCCGACCATTACCAACAAAATACCCCAATAGGTGACGGGCCTGTCCTACTTCCTGACAATCATTATCTAAGTACCCAGTCGGCCCTATCGAAGGACCCTAATGAGAAACGTGATCATATGGTGCTTCTAGAATTCGTCACCGCCGCAGGGATAACCCTAGGGATGGACGAGCTATATAAGTAA

>GFP_S5

ATGGTCTCGAAGGGGGAAGAACTATTCACCGGGGTGGTGCCTATACTAGTGGAACTAGACGGGGACGTGAATGGGCATAAATTTTCGGTCTCGGGGGAAGGGGAAGGGGATGCCACCTATGGTAAACTAACCCTAAAATTCATATGCACCACCGGTAAGCTACCAGTCCCTTGGCCTACCCTTGTGACCACCCTTACATATGGGGTCCAATGCTTCTCGCGTTATCCTGACCATATGAAACAACATGATTTTTTTAAATCGGCCATGCCTGAGGGTTATGTCCAAGAAAGAACAATATTCTTCAAAGACGACGGGAACTATAAAACCCGTGCCGAAGTCAAATTCGAAGGGGACACACTTGTCAATAGAATTGAACTTAAAGGGATAGACTTCAAGGAGGACGGGAATATACTAGGGCATAAGCTAGAATATAATTACAACTCGCATAATGTGTATATAATGGCAGATAAACAAAAAAACGGGATAAAAGTCAACTTCAAGATAAGACATAATATAGAAGACGGTTCGGTCCAGCTTGCCGACCATTATCAACAAAATACACCTATAGGTGACGGGCCAGTCCTTCTACCTGATAATCACTATCTTAGTACACAGTCGGCACTTAGTAAGGACCCAAATGAAAAAAGAGACCACATGGTCCTTCTAGAATTCGTCACCGCCGCCGGGATTACCCTAGGGATGGATGAACTATATAAATAA

>GFP_p1

ATGGTGAGTAAGGGTGAGGAGCTTTTTACAGGTGTGGTGCCAATTCTTGTGGAGCTTGATGGTGATGTGAACGGTCACAAGTTTAGTGTGAGTGGTGAGGGTGAGGGTGATGCAACATACGGTAAGCTTACACTTAAGTTTATTTGCACAACAGGTAAGCTTCCAGTGCCATGGCCAACACTTGTGACAACACTTACATACGGTGTGCAGTGCTTTAGTCGTTACCCAGATCACATGAAGCAGCACGATTTTTTTAAGAGTGCAATGCCAGAGGGTTACGTGCAGGAGCGTACAATTTTTTTTAAGGATGATGGTAACTACAAGACACGTGCAGAGGTGAAGTTTGAGGGTGATACACTTGTGAACCGTATTGAGCTTAAGGGTATTGATTTTAAGGAGGATGGTAACATTCTTGGTCACAAGCTTGAGTACAACTACAACAGTCACAACGTGTACATTATGGCAGATAAGCAGAAGAACGGTATTAAGGTGAACTTTAAGATTCGTCACAACATTGAGGATGGTAGTGTGCAGCTTGCAGATCACTACCAGCAGAACACACCAATTGGTGATGGTCCAGTGCTTCTTCCAGATAACCACTACCTTAGTACACAGAGTGCACTTAGTAAGGATCCAAACGAGAAGCGTGATCACATGGTGCTTCTTGAGTTTGTGACAGCAGCgGGTATTACACTTGGTATGGATGAGCTTTACAAGTAA

>GFP_p2

ATGGTTTCGAAAGGTGAGGAGCTCTTCACCGGTGTTGTTCCGATCCTCGTTGAGCTCGACGGTGACGTTAACGGTCATAAATTCTCGGTTTCGGGTGAGGGTGAGGGTGACGCAACCTACGGTAAACTCACCCTCAAATTCATCTGCACCACCGGTAAACTCCCGGTTCCGTGGCCGACCCTCGTTACCACCCTCACCTACGGTGTTCAATGCTTCTCGAGATACCCGGACCATATGAAACAACATGACTTCTTCAAATCGGCAATGCCGGAGGGTTACGTTCAAGAGAGAACCATCTTCTTCAAAGACGACGGTAACTACAAAACCAGAGCAGAGGTTAAATTCGAGGGTGACACCCTCGTTAACAGAATCGAGCTCAAAGGTATCGACTTCAAAGAGGACGGTAACATCCTCGGTCATAAACTCGAGTACAACTACAACTCGCATAACGTTTACATCATGGCAGACAAACAAAAAAACGGTATCAAAGTTAACTTCAAAATCAGACATAACATCGAGGACGGTTCGGTTCAACTCGCAGACCATTACCAACAAAACACCCCGATCGGTGACGGTCCGGTTCTCCTCCCGGACAACCATTACCTCTCGACCCAATCGGCACTCTCGAAAGACCCGAACGAGAAAAGAGACCATATGGTTCTCCTCGAGTTCGTTACCGCAGCAGGTATCACCCTCGGTATGGACGAGCTCTACAAATAA

>GFP_p3

ATGGTGTCGAAGGGCGAAGAACTGTTCACGGGCGTGGTGCCGATCCTGGTGGAACTGGACGGCGACGTGAACGGCCACAAGTTCTCGGTGTCGGGCGAAGGCGAAGGCGACGCAACGTACGGCAAGCTGACGCTGAAGTTCATCTGCACGACGGGCAAGCTGCCGGTGCCGTGGCCGACGCTGGTGACGACGCTGACGTACGGCGTGCAATGCTTCTCGCGCTACCCGGACCACATGAAGCAACACGACTTCTTCAAGTCGGCAATGCCGGAAGGCTACGTGCAAGAACGCACGATCTTCTTCAAGGACGACGGCAACTACAAGACGCGCGCAGAAGTGAAGTTCGAAGGCGACACGCTGGTGAACCGCATCGAACTGAAGGGCATCGACTTCAAGGAAGACGGCAACATCCTGGGCCACAAGCTGGAATACAACTACAACTCGCACAACGTGTACATCATGGCAGACAAGCAAAAGAACGGCATCAAGGTGAACTTCAAGATCCGCCACAACATCGAAGACGGCTCGGTGCAACTGGCAGACCACTACCAACAAAACACGCCGATCGGCGACGGCCCGGTGCTGCTGCCGGACAACCACTACCTGTCGACGCAATCGGCACTGTCGAAGGACCCGAACGAAAAGCGCGACCACATGGTGCTGCTGGAATTCGTGACGGCAGCAGGCATCACGCTGGGCATGGACGAACTGTACAAGTAA

>GFP_163

atggtgagcaagggggaggagctgttcaccggggtggtgcccatcctggtcgagctggacggcgacgtaaacggccacaagttcagcgtgtccggcgagggcgagggcgatgccacctacggcaagctgaccctgaagttcatctgcaccaccggcaagctgcccgtgccctggcccaccctcgtgaccaccctgacctacggggtgcagtgcttctctagatacccggaccacatgaaacagcacgatttcttcaaaagcgcgatgcccgagggatacgttcaagaaagaaccatcttcttcaaagatgatggtaactacaaaacacgcgcggaggtaaagttcgagggcgacactctggtaaacagaatcgaattgaaaggtatcgacttcaaagaagacggtaacattctcggccacaaattagaatacaactacaactcccacaacgtttacatcatggcagacaaacagaaaaacggcatcaaagttaacttcaaaatcagacacaacatcgaagacggtagcgtacagctggccgatcactaccagcagaacactccgatcggcgacggcccggtactgttgccggacaaccactacctgtctacccagtcggccctgtccaaagacccgaacgaaaaaagagatcacatggttctgctggagttcgtaaccgcagctggcatcactctgggcatggacgaactgtacaaataa

>GFP_205

atggtgagcaaaggtgaggagctgttcaccggcgtggtgcccatcctcgtcgagttagacggcgacgtcaacgggcacaagttcagcgtgtcgggcgagggcgagggggatgcaacatacgggaaattgacgctcaagttcatctgcaccacgggcaagctcccggtcccctggcccaccctcgtcacgaccctgacctacggtgtccagtgcttctctagatatcccgatcatatgaagcagcatgacttcttcaaaagtgctatgcccgaaggctacgtccaagagaggacgatatttttcaaggatgatgggaattacaagacacgggcggaggtgaagttcgagggggatactctagtgaacagaattgaattgaaaggaattgacttcaaagaagacggcaacatactcggccacaaattggaatataattacaattcccacaatgtatacattatggccgataaacagaagaacggcatcaaagttaacttcaaaatcagacacaacatcgaagacggtagcgtacagctggccgatcactaccagcagaacactccgatcggcgacggcccggtactgttgccggacaaccactacctgtctacccagtcggccctgtccaaagacccgaacgaaaaaagagatcacatggttctgctggagttcgtaaccgcagctggcatcactctgggcatggacgaactgtacaaataa

>GFP_211

atggtgagcaaaggtgaggagctgttcaccggcgtggtgcccatcctcgtcgagttagacggcgacgtcaacgggcacaagttcagcgtgtcgggcgagggcgagggggatgcaacatacgggaaattgacgctcaagttcatctgcaccacgggcaagctcccggtcccctggcccaccctcgtcacgaccctgacctacggtgtccagtgcttctctagataccccgaccacatgaagcagcacgacttctttaaaagtgctatgcccgagggatacgttcaggagaggacgattttcttcaaggacgacggcaactacaagacgcgtgcggaggtaaagttcgagggagacacgctcgtgaacaggatagagttgaaggggatcgacttcaaggaggacgggaacatactcggccacaagttggagtacaactacaactcgcacaacgtctacatcatggcggataagcagaaaaatggtataaaagttaacttcaagatcaggcacaacatcgaggacgggagtgtgcagcttgcagaccactaccagcagaacacgcccatcggcgacggacccgtcctcttgccggacaaccactacctgtctacccagtccgcgctctcgaaggaccccaatgaaaaaagagatcatatggttcttttagagttcgttacagcagcaggcatcacgctaggtatggatgagctttataaataa

>GFP_224

atggtgagcaaaggtgaggagctgttcaccggcgtggtgcccatcctcgtcgagttagacggcgacgtcaacgggcacaagttcagcgtgtcgggcgagggcgagggggatgcaacatacgggaaattgacgctcaagttcatctgcaccacgggcaagctcccggtcccctggcccaccctcgtcacgaccctgacctacggtgtccagtgcttctctagatatcccgatcatatgaagcagcatgacttcttcaaaagtgctatgcccgaaggctacgtccaagagaggacgatatttttcaaggatgatgggaattacaagacacgggcggaggtgaagttcgagggggatactctagtgaacagaattgaattgaaaggaattgacttcaaagaagacggcaacatactcggccacaaattggaatataattacaattcccacaatgtatacattatggccgataaacagaagaacggcatcaaagttaacttcaaaatcagacacaacatcgaagacggtagcgtacagctggccgatcactaccagcagaacactccgatcggcgacggcccggtactgttgccggacaaccactacctgtctacccagtcggccctgtccaaagacccgaacgaaaaaagagatcacatggttctgctggagttcgtaaccgcagctggcatcactctgggcatggacgaactgtacaaataa

>GFP_065

ATGGTCAGCAAAGGCGAGGAACTCTTCACCGGAGTCGTCCCTATCCTCGTGGAACTGGACGGAGACGTGAACGGCCACAAGTTCAGCGTGTCCGGCGAGGGGGAGGGAGATGCAACTTACGGTAAACTCACACTCAAGTTCATTTGCACTACAGGGAAGCTCCCGGTGCCGTGGCCCACCCTCGTGACCACGCTCACTTATGGTGTGCAGTGCTTTTCCCGTTATCCCGACCACATGAAGCAGCATGACTTCTTTAAATCCGCGATGCCCGAGGGGTATGTGCAGGAGCGCACCATCTTCTTCAAGGACGACGGTAACTATAAAACAAGGGCAGAGGTTAAATTCGAGGGGGACACGCTCGTCAACCGTATTGAGCTCAAAGGCATAGACTTCAAGGAGGACGGAAACATTCTCGGTCACAAGCTCGAGTATAACTACAACTCCCACAACGTCTACATTATGGCAGACAAACAAAAGAACGGCATTAAGGTGAACTTCAAGATCCGCCATAACATAGAGGACGGCAGCGTCCAACTCGCGGACCACTACCAACAGAACACGCCCATTGGGGACGGACCCGTGCTGCTCCCTGACAATCATTATCTGTCCACTCAGTCCGCTCTCAGCAAGGACCCCAACGAGAAACGTGACCATATGGTGCTCCTCGAGTTCGTGACAGCCGCCGGGATAACGCTCGGTATGGACGAGCTCTATAAAtaa

>GFP_075

ATGGTGTCCAAAGGCGAGGAGCTCTTCACAGGAGTCGTGCCCATTCTCGTCGAGCTCGACGGTGACGTTAACGGTCACAAGTTCTCCGTCAGCGGAGAGGGTGAGGGCGACGCAACGTACGGCAAGCTCACACTCAAATTTATTTGCACGACTGGTAAACTCCCCGTTCCCTGGCCTACCCTCGTGACCACACTCACGTACGGAGTGCAGTGCTTCTCCCGTTACCCGGACCACATGAAACAGCACGACTTCTTTAAATCCGCCATGCCGGAGGGCTATGTGCAGGAGCGTACAATCTTCTTCAAGGACGATGGTAACTATAAAACACGTGCTGAGGTCAAGTTCGAGGGGGACACCCTGGTTAACCGTATCGAGCTCAAGGGGATCGACTTCAAAGAGGACGGCAACATTCTGGGTCACAAACTCGAGTATAACTACAACAGCCACAACGTCTATATTATGGCCGACAAGCAGAAAAACGGAATAAAGGTAAACTTTAAGATACGCCATAACATCGAGGACGGCTCCGTGCAGCTCGCTGACCACTATCAACAGAACACTCCCATTGGTGACGGCCCCGTCCTCCTCCCGGACAACCACTACCTCAGCACACAGAGCGCACTCAGCAAAGACCCCAACGAGAAACGCGACCATATGGTGCTCCTCGAGTTCGTCACCGCCGCAGGGATCACGCTCGGGATGGACGAGCTCTATAAAtaa

>GFP_095

ATGGTGTCCAAGGGAGAGGAGCTCTTCACAGGAGTTGTCCCCATTCTCGTCGAGCTGGACGGCGACGTCAACGGACACAAGTTCAGCGTGAGCGGAGAGGGCGAGGGAGACGCTACGTACGGAAAACTCACACTCAAGTTCATCTGCACCACAGGCAAGCTCCCGGTGCCCTGGCCCACGCTCGTCACCACGCTCACGTATGGCGTGCAGTGCTTTAGCCGTTATCCGGACCACATGAAGCAGCACGACTTCTTCAAAAGCGCCATGCCGGAGGGCTACGTCCAGGAGCGCACGATTTTCTTCAAGGACGACGGTAACTATAAGACACGTGCCGAGGTCAAGTTCGAGGGCGACACGCTCGTGAACCGCATCGAGCTGAAGGGTATCGACTTCAAAGAGGACGGTAACATTCTCGGTCACAAGCTCGAGTACAACTATAACTCCCACAACGTGTACATTATGGCAGACAAGCAGAAGAACGGCATTAAAGTGAACTTCAAAATCCGTCACAACATTGAGGACGGCTCCGTCCAGCTCGCAGACCACTATCAGCAGAACACGCCGATAGGTGACGGCCCCGTCCTCCTCCCGGACAACCACTACCTCAGCACACAGAGCGCTCTCTCTAAAGACCCCAACGAGAAACGTGACCACATGGTGCTCCTCGAGTTCGTCACAGCAGCTGGCATTACGCTCGGTATGGACGAACTCTACAAAtaa
